# Molecular Mechanisms of the Binding and Specificity of *Streptococcus Pneumoniae* Sortase C Enzymes for Pilin Subunits

**DOI:** 10.1038/s41598-017-13135-3

**Published:** 2017-10-13

**Authors:** Emmanuel B. Naziga, Jeff Wereszczynski

**Affiliations:** Department of Physics and Center for Molecular Study of Condensed Soft Matter, Illinois Institute of Technology, 3440 S Dearborn St, Chicago, IL 60616 USA

## Abstract

Pili are elongated structures that protrude from bacteria and increase their virulence. The *Streptococcus pnuenomae* pilus island 1 pili are composed of three subunits, RrgA, RrgB, and RrgC, and are assembled by three class C sortase C (SrtC) enzymes: SrtC-1, SrtC-2, and SrtC-3. Pilin subunits are recognized by SrtC proteins through a pentapeptide sorting signal, and while previous studies have sought to characterize the selectivities of SrtC isoforms for these subunits, the molecular mechanisms underlying these interactions remain unclear. Here, we report a series of molecular dynamics simulations of each SrtC enzyme with the sorting signals of RrgA, RrgB, and RrgC to determine the structural and thermodynamic basis of pilin recognition. Results show that, in accordance with previous studies, both SrtC-1 and SrtC-3 are selective for RrgB, while SrtC-2 is selective for RrgA. This specificity is tuned by the sorting signal binding conformation in which the first two residue sidechains complement hydrophobic residues around the active site, while the third residue projects away from the catalytic triad and makes specific interactions based on its charge and reach. Together, these results provided atomic-scale descriptions of the SrtC substrate selectivity mechanisms and extend the emerging model of pilin construction in *S. pnuenomae*.

## Introduction

The human pathogen *Streptococcus pneumoniae* is an infectious agent responsible for over 1.6 million deaths worldwide a year, largely among the young and the elderly, and is the leading cause of multiple diseases, including bacterial pneumonia, sepsis, and meningitis^1–3^. The virulence of these Gram-positive bacteria is increased by pili, elongated fibrous structures on their surface that mediate intercellular adhesion during the colonization process^4^. Although not all *S*. *pneumoniae* serotypes rely on pili, pneumococci clones that are nonsusceptible to penicillin tend to have an increased reliance on them for their virulence^5^. Therefore, this direct link between pili formation and infection presents an exciting new antibacterial target for strains that are resistant to conventional therapeutics^6,7^.

*S*. *pneumoniae* express two types of pili^8^. Approximately 30% of strains contain pilus island 1 (PI-1) pili, which are constructed of an elongated shaft of the main pilin subunit RrgB that is decorated with the minor pilin subunit RrgA and anchored to the cell wall by RrgC^4,9,10^. The architects of pili are class C Sortase enzymes (SrtC), membrane-associated cysteine transpeptidases that covalently link subunits to one another and are necessary and sufficient for pili construction^9,11–13^. SrtC functions by specifically recognizing a conserved LPXTG-like pentapeptide sorting signal motif in the appropriate pilin subunit, forming an acyl-enzyme intermediate to this first substrate, and then catalyzing its covalent linkage to another pilin subunit, the second substrate of catalysis^14,15^.

In *S*. *pneumoniae*, the rlr-A islet encodes for the three PI-1 pilin subunits as well as the three SrtC isoforms that participate in their assembly: SrtC-1, SrtC-2, and SrtC-3^8^. The roles for each of these sortases remains unclear, however it is believed that their function is dependent on their differing affinities for each of the three subunits^16,17^. For their first substrate, SrtC-1 is selective for the backbone unit RrgB, SrtC-2 is selective for the accessory subunit RrgA, and SrtC-3 is selective for RrgB, although it does not appear to have as strong an affinity as between SrtC-1 and RrgB^18^. It is unclear what affinity, if any, these sortases have for the cell wall anchoring subunit RrgC^4,16^, and in fact it has recently been suggested that RrgC is recognized not by a SrtC enzyme but by the housekeeping sortase A (SrtA) protein^19^. Experiments in which the sorting signal was swapped between subunit types have demonstrated that SrtC selectivity is primarily due to the sorting signal sequence, which have only slight differences from one another (RrgA: YPRTG, RrgB: IPQTG, RrgC: VPDTG), although their net charges do differ at physiological pH^17^.

**Figure 1:**
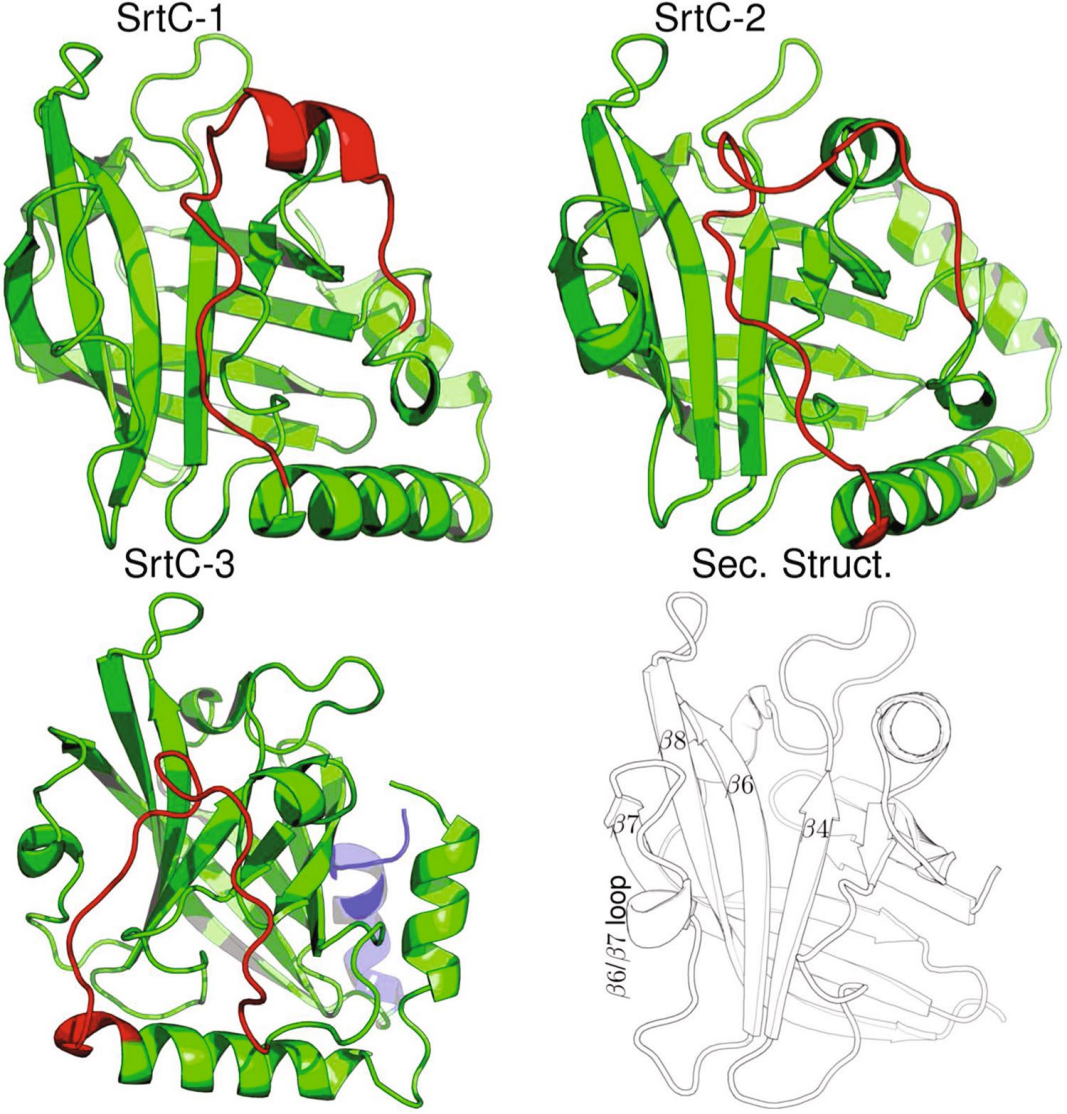
Representations of the three sortase C enzymes considered in this study. The lid region of the enzymes is colored in red while the remainder are in green except the C-terminal helix of SrtC-3 that is colored in blue for emphasis. The names of structural elements are shown in the picture in the bottom right-hand side.

Structural studies have shown that all SrtC enzymes adopt an overall fold centered around an eight-stranded β-barrel and an active-site consisting of the conserved Cys-His-Arg catalytic triad, similar to the more widely studied housekeeping Sortase A (SrtA) enzymes (Fig. 1)^16,20^. However, SrtC structures also contain significant differences from SrtA, such as additional α-helices, and an N-terminal region which can be either a loop or a helix and is anchored in the active site and creates a “lid”^21^. Since the lid typically covers the active site, it is believed that it must “open” to allow substrate binding, and thus serves as a regulator of pili construction^22^. In previous work^23^, we showed that despite the prevailing hypothesis that the lid of SrtC-1 is flexible in solution, it is remarkably rigid in the absence of a substrate and that the free energy of lid opening is prohibitive, suggesting that this process is likely assisted by a hitherto unknown factor *in vitro*.

There has yet to be an experimentally resolved structure of a substrate-bound/SrtC enzyme. However, substrate bound structures exist for two others sortases, the *Staphylococcus aureus* class A and class B sortases (SrtA and SrtB)^24,25^. These structures, which contain sorting signals LPATG and NPQTG (respectively) covalently bound to the active site cysteine residue, show two distinct orientations which are characterized by the threonine residue position. In the Thr-In conformation observed in SrtB, the sidechain of the sorting signal threonine is pointed towards the conserved active site arginine that is part of the catalytic triad, while the sidechain of the residue upstream of the threonine is solvent exposed. On the other hand, in the Thr-Out conformation observed in SrtA, the sorting signal threonine is solvent exposed while the upstream residue (alanine) points inward to the binding pocket. Free energy calculations revealed that Thr-In and Thr-Out are both accessible for this SrtA complex, while Thr-In is the dominant conformation in the SrtB system. However, given that the Thr-In conformation positions the catalytically critical sorting signal threonine so that it can be recognized by the sortase and facilitates the creation of an oxyanion hole, and taken with the fact that hybrid QM/MM simulations have suggested a lower free energy barrier for the acylation reaction in Thr-In, the Thr-In conformation is believed to be the more catalytically relevant state^25,26^.

To determine the molecular mechanisms underlying substrate binding and specificity by SrtC enzymes, and in particular for the isoforms of *S*. *pneumoniaeS*. *pneumoniae*, we have performed a series of molecular dynamics (MD) simulations of both substrate free (apo) and bound (holo) SrtC-1, SrtC-2, and SrtC-3 enzymes. Endpoint based energy decomposition analyses were performed for each of the nine potential sortase/substrate complexes, as well as more rigorous alchemical calculations to compute the relative association free energies of Rrg sorting signals to each SrtC enzyme. The results reveal details of specific residue interactions in the active site and surrounding loops that results in sorting signal selectivity. The size, and hence reach, of the middle residue in the five-residue sorting signal plays a vital role in the interaction with SrtC and strongly influences binding preferences. Finally, binding free energy calculations shed light on the relative affinities for each SrtC isoform with different sorting signals, and have implications for our understanding of the mechanism of pilin assembly.

## Methods

### Apo System Simulations

Starting structures for SrtC-1, SrtC-2, and SrtC-3 were obtained from the protein databank structures 2WIJ^27^, 3G66^20^, and 2W1K^27^, respectively. Residues Glu^91^ and Gln^92^, which were missing in the SrtC-2 structure, were added to the initial structure using the *modeller* program. These structures were then processed using the *Leap* module of the AMBER software suite^28^ to add hydrogens and solvated in a box of TIP3P^29^ water molecules with a minimum distance of 12 Å from any protein atom to the edge of the box. Additional ions were added to create an ~150 mM NaCl environment. Structures were minimized and equilibrated through a multi-stage protocol. First, solvent molecules were minimized while the protein atoms were fixed for 15,000 steps, which was followed by 10,000 minimization steps where protein atoms were free. Next, the temperature of the system was increased from 0 to 300 K during a 200 ps constant volume molecular dynamics (MD) calculation. Finally, constant pressure MD simulations of 1.0 μs were performed. A 2 femtosecond timestep was used for all MD calculations. Van der Waals and direct Coulomb interactions were truncated at 10 Å, while long-range electrostatics were treated with particle mesh Ewald summation calculations using a maximum grid spacing of 1 Å and a fourth order spline^30^. The AMBER FF14SB forcefield^31^, along with the GPU accelerated version of PMEMD^32,33^, were used for all calculations except the alchemical simulations described below which used the CPU implementation since this functionality has yet to be implemented in the GPU version. Simulations were performed on local resources as well as The Extreme Science and Engineering Development Environment (XSEDE)^34^.

### Holo Complex Simulations

Holo complex models were developed based on the 1.0 μs equilibrated apo structures and the experimentally determined Thr-In sorting signal/Sortase B (SrtB) structure^25^. The proposed (see Introduction) primary sorting signal for each sortase was used as a substrate to model each system. Specifically, SrtC-1 and SrtC-3 were complexed with the IPQTG sorting signal of RrgB while the YPRTG sorting signal of RrgA was modelled into SrtC-2. These complexes were made using VMD by aligning the respective sortase C into the crystal structure of SrtB in complex with its NPQTG sorting signal^25^. Sorting signal residues were then mutated as appropriate. The resulting structures were then solvated in water and equilibrated as described above for the apo state and a 1.0 μs NPT simulation was performed for each complex.

### MD Calculations for MM/GBSA and Decomposition Analysis

To examine the binding energetics of all three sorting signals to each of the three sortase C enzymes, further MD calculations were implemented starting from the equilibrated complexes described in the previous section. Water molecules were stripped from the resulting equilibrated structure and the sorting signals were mutated as necessary. For instance, the SrtC-1 + IPQTG complex was mutated to SrtC-1 + YPRTG and SrtC-1 + VPDTG complexes, while a similar procedure was carried out for SrtC-2 and SrtC-3, resulting in a total of nine systems. Each was then solvated, minimized and heated to 300 K as previously described. However, instead of one long constant pressure calculation, 50 independent MD calculations of 10 ns duration were implemented for each system, with the first 1 ns discarded before analysis was performed^35^. This resulted in a total of 500 ns of simulation time for each complex. All three sorting signals were also individually solvated and simulated for 3.0 μs using the same procedures described above for the apo systems. The three-trajectory approach with independent MD simulations for the complex, receptor, and ligand was used for the MM/GBSA and energy decomposition analysis^36,37^. 1000 snapshots were used for MM/GBSA analysis which corresponds to one sample per 300 ps, and is beyond our calculated statistical efficiency of 150 ps. The Generalized Born implicit solvent model using the atomic radii developed by Onufriev, Bashford and Case^38,39^ was used for the analysis. The non-polar contribution to solvation was calculated using the linear combination of pairwise overlaps method with a probe radius of 1.4 Å. The MMPBSA.py script was used for this analysis^40^.

### Thermodynamic Integration

We also performed relative alchemical calculations that mutated sorting signals from one pilin type to another^41,42^. Specifically, an initial SrtC-1 + IPQTG complex was mutated to SrtC-1 + YPRTG and SrtC-1 + VPDTG, while the same procedure was carried out for SrtC-2 + IPQTG and SrtC-3 + IPQTG complexes. A three-step protocol of charge removal, Van der Waals and bonded transformation, and charge addition was implemented. During the calculations, both initial residues (e.g. Ile and Gln of IPQTG) were simultaneously transformed to the appropriate target residue. Eleven windows were used for each transformation step, with the λ parameter varying from 0.0 to 1.0 in 0.1 increments. A 12 ns MD calculation was run in each window and the first 4 ns discarded as equilibration, for a total of 396 ns of simulation per complex, with 264 ns used for analysis in each TI calculation. Alchemical calculations were also performed for transformation of the sorting signals in solvent following an identical protocol. All calculations were performed three times to improve convergence^43^. Free energy differences were estimated with thermodynamic integration (TI) trapezoid and cubic trapezoid integration^44^, Bennet acceptance ratio (BAR)^45^, and multistate Bennet acceptance ratio (MBAR) calculations^46^, as implemented in the Alchemical-analysis.py script of Mobley and coworkers^47^. The BAR and trapezoid methods yielded almost identical results, therefore results from the trapezoid method are presented.

### Clustering and Other Analyses

For cluster analysis, the MD structures were aligned to the Cα atoms of residues in the β-barrel portion of the protein (e.g. residues 81 to 171 and 186 to 204 in SrtC-1) of the receptor (SrtC) since they are the least dynamic. Thereafter, clustering was performed based on the RMSD of the sorting signal heavy atoms. The hierarchical agglomerative algorithm with single-linkage was used for the analysis with a minimum distance (RMSD) of 1.0 Å between clusters. Hydrogen bonding interactions were determined to be present at a receptor/donor distance of 3.5 Å and an angle greater than 135°. Dynamics of the apo and holo states were compared with Kullback-Liebler divergence analysis using the script developed by Jacobson and coworkers^29^. Standard error of the mean values was calculated by dividing computed standard deviation values by the number of independent data points. Figures were made with VMD^48^ and PyMOL^49^.

## Results

Binding Conformations and Dynamics of Rrg Sorting Signals in Complex with Sortase C Proteins

**Figure 2:**
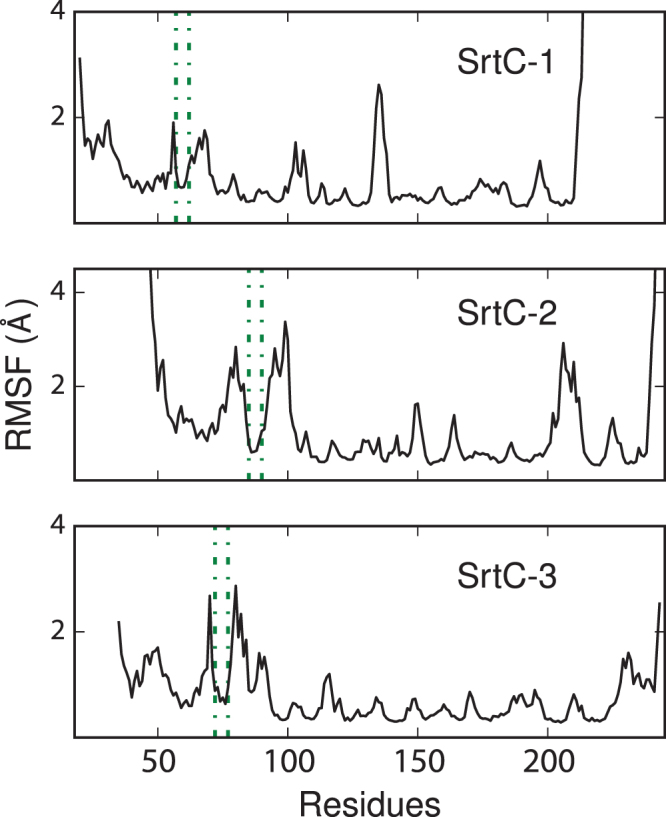
Root mean squared fluctuations for the apo state of the SrtC-1 (top), SrtC-2 (middle) and SrtC-3 (bottom) proteins. The positions corresponding to the lid anchor regions are indicated by the dashed green lines. The hinge residues on both side of the dashed lines show elevated dynamics.

Previous computational and experimental results have indicated that, in solution, the lid region of SrtC-1 remains in a closed conformation that occludes the enzyme’s active site^25^. The MD simulations of the apo SrtC-2 and SrtC-3 enzymes performed here suggest that the lid in these isoforms have similar stabilities to the lid in SrtC-1. In this region (between the dotted lines in Fig. 2), the root means squared fluctuations (RMSF) for SrtC-2 and SrtC-3 are similar to SrtC-1, with values of less than 1 Å for residues forming the salt-bridge interactions that anchors the lid into the active site for each system. However, as noted previously, the residues on both sides of the anchoring residues have significantly higher RMSFs, which has led to previous proposals that they constitute hinges for lid opening^27^. In addition, each sortase possesses hydrophobic residues in the lid region preceding the anchor residues that cover a hydrophobic nook at the base of the β6/β7 loop opposite the lid, which remain stable in our simulations. These results suggest that a closed lid blocking this hydrophobic nook and the active site, and requiring an external factor for opening, may be a common feature found in lid bearing class C sortases.

Given that no experimentally resolved SrtC/substrate structure has been reported, models of the substrate bound complexes were constructed based on the Thr-In state of SrtB (see Methods). We initially considered models of SrtC-1 in complex with the RrgB (IPQTG), SrtC-2 with the RrgA (YPRTG), and SrtC-3 with the RrgB (IPQTG) sorting signals, which correspond to the putative binding partners in the literature^16–18^. These binding modes were stable over the course of 1.0 μs of simulation, suggesting that the models represent stable binding states (Figure S1). To further characterize the binding modes, data shown in Table 1 details the hydrogen bonding interactions observed between the RrgB sorting signal (IPQTG) and various residues in and around the active sites of SrtC-1. The most stable interaction was between the sorting signal isoleucine backbone and Arg^202^ in the catalytic triad, which occurred for 82% of the simulation. The backbone nitrogen of the sorting signal isoleucine interacted with the backbone of Pro^173^, which is in the loop opposite the lid, while the proline also formed a hydrogen bond to Arg^202^. Other interactions occurred between the sorting signal threonine and glycine and Thr^132^ that is in the loop adjacent to the active site.

**Table 1:** Hydrogen bonding interactions observed in MD simulations of SrtC-1, SrtC-2, and SrtC-3 and their experimentally proposed sorting signal partners.

SrtC-1 + IPQTG			SrtC-2 + YPRTG			SrtC-3 + IPQTG		
Sorting Signal	Sortase	%	Sorting Signal	Sortase	%	Sorting Signal	Sortase	%
Ile	Arg^202^	82	Tyr	Asp^203^	48	Ile	Arg^215^	52
Ile	Pro^173^	57	Pro	Arg^230^	62	Pro	Arg^215^	38
Pro	Arg^202^	13	Arg	Glu^130^	88	Gln	Ala^113^	23
Gln	Asn^199^	13	Arg	Glu^200^	36	Gln	Arg^215^	12
Gln	Thr^132^	24	Thr	Glu^130^	125			
Thr	Thr^129^	23	Thr	Arg^230^	6			
Thr	Thr^132^	53	Gly	Arg^230^	26			
Gly	Thr^132^	24						

The percentage occupancies for hydrogen bond donating and accepting groups of a particular residue are summed together, therefore, it is possible to have numbers greater than 100%.

**Figure 3:**
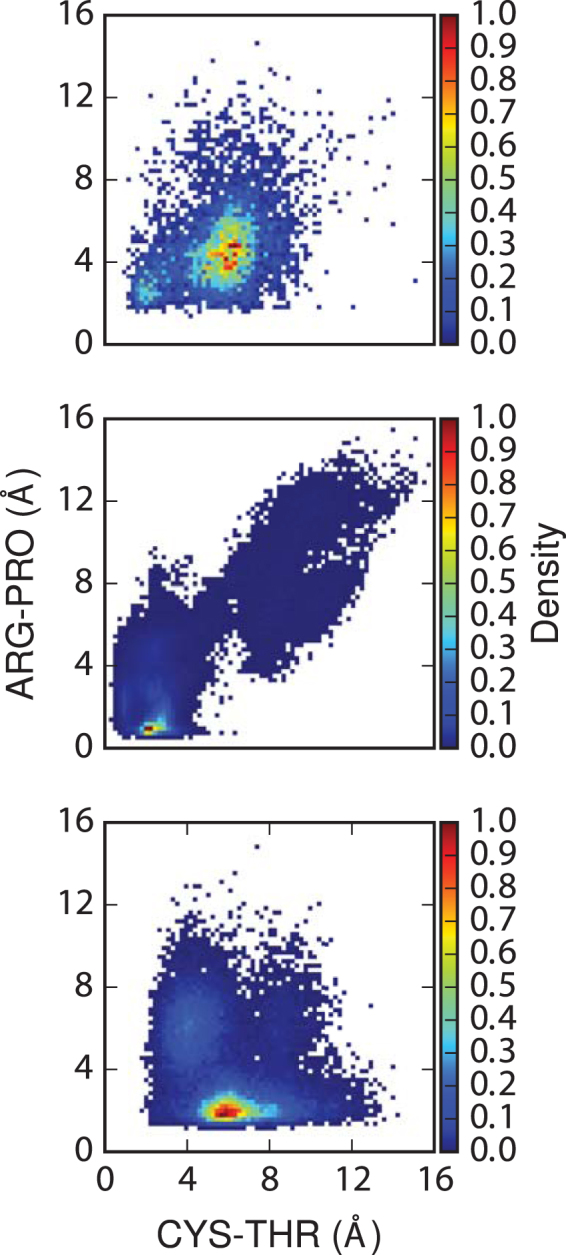
Distances between the catalytic cysteine residue (Sulphur atom) and sorting signal threonine (backbone carbon) versus the active site arginine to sorting signal proline for the SrtC-1 + IPQTG (top), SrtC2 + YPRTG (middle) and SrtC-3 + IPQTG (bottom) complexes.

**Figure 4:**
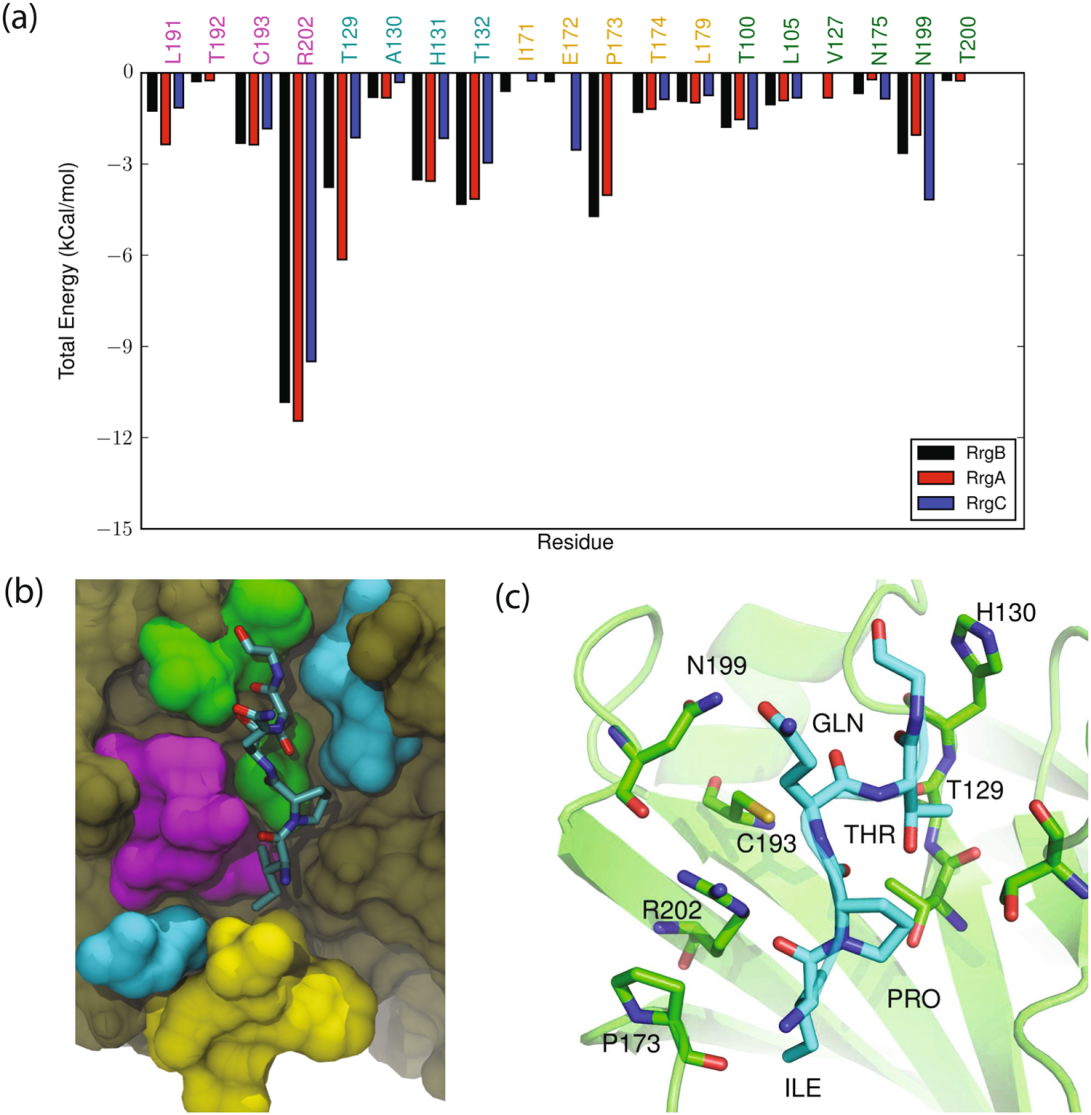
Interactions of Rrg sorting signals with SrtC-1: (**a**) Comparison of the total interaction energies between the RrgA (YPQTG), RrgB (IPQTG) and RrgC (VPDTG) sorting signals and the SrtC-1 protein. The four regions of SrtC-1 that interact with the sorting signal as described in the text are color coded with residue names in the graph, (**b**) Color coded surface representation of the aforementioned interaction regions and (**c**) Binding pose of the RrgB (IPQTG) sorting signal in the active site of SrtC-1 as obtained from cluster analysis. The sorting signal is shown in licorice representation with its backbone additionally drawn as a yellow tube. Some important sortase residues that interact with the sorting signal are shown in ball and stick representation with associated one letter code and residue number.

The sorting signal binding states were further characterized through a clustering analysis. To represent the different substrate binding modes, the MD structures were aligned to the Cα atoms of residues in the β-barrel (residues 81 to 171 and 186 to 204, in SrtC-1) and then clustering was performed based on the RMSD of the sorting signal heavy atoms. The cluster analysis produced few dominant clusters, with the first two clusters representing 65% and 15% of the simulation data, respectively. However, many other clusters, most with a single MD frame were obtained. The average distance (RMSD) of cluster one from all other clusters is approximately 2.1 Å, indicating that these structures are not significantly different. Examination of the structures in the dominant clusters reveals that the main difference between these two clusters are in the last two residues and that they generally represent two orientations of the same binding pose, which is reflected in a RMSD of approximately 1.3 Å between their cluster representative structures (Table S1). This binding conformation provides several stabilizing interactions for sorting signal attachment. First, the hydrophobic sidechains of the isoleucine and proline point towards the hydrophobic nook that is located towards the end of the active site groove, which contains the sidechains of Leu^112^, Val^127^, Leu^152^, Phe^171^, Leu^179^, Leu^191^, and Leu^177^. Insertion of the sorting signal into this region in the holo complexes replaces some of the hydrophobic contacts formed in the apo structure by the hydrophobic portions of the lid that are displaced by substrate binding. Additionally, this binding pose places the backbone of the isoleucine and proline in contact with the active site Arg^202^ and Pro^173^ in the β6/β7 loop (Table 1 and Figs 3 & 4). The sidechain of the glutamine projects out of the active site and interacts with Asn^199^ in the β7/β8 loop. While there are differences between the orientations of the threonine and glycine in the structures in the top two clusters, hydrogen bonding by these two residues and those in the surrounding loop contribute to stabilizing the sorting signal in the active site groove. Moreover, these interactions maintain close contacts between residues in the catalytic triad and the sorting signal. The average Cys^193^ Sulphur –Thr C_α_ and Arg^202^-Pro distances were 5.9 ± 1.0 Å and 3.8 ± 0.7 Å respectively, helping to maintain the catalytic residues in a conformation necessary for the nucleophilic attack required for the first step of the transpeptidation reaction.

**Figure 5:**
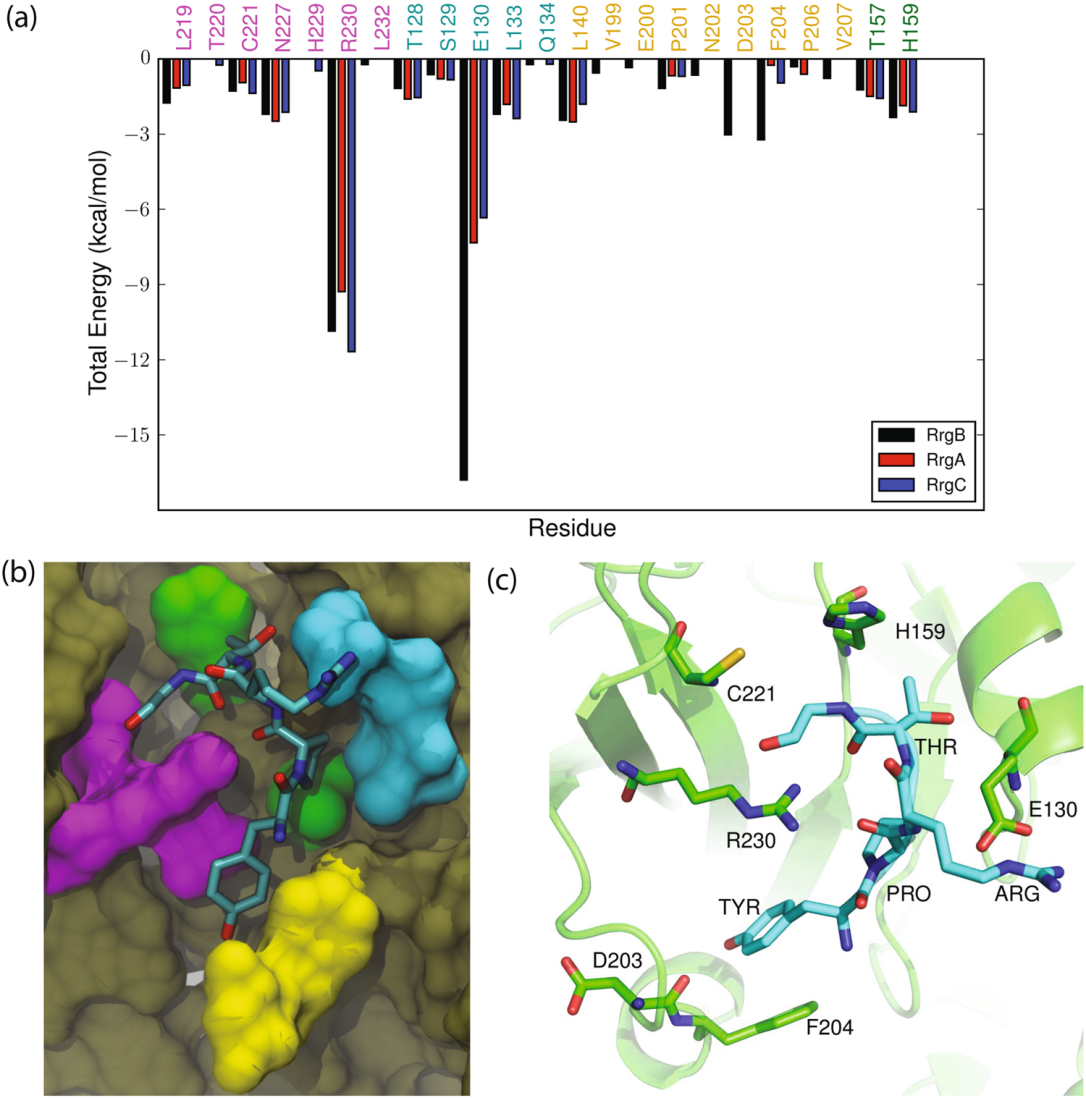
Interactions of Rrg sorting signals with SrtC-2: (**a**) Comparison of the total interaction energies between the RrgA (YPQTG), RrgB (IPQTG) and RrgC (VPDTG) sorting signals and the SrtC-2 protein. The four regions of SrtC-2 that interact with the sorting signal as described in the text are color coded with residue names in the graph, (**b**) Color coded surface representation of the aforementioned interaction regions and (**c**) Binding pose of the RrgA (YPRTG) sorting signal in the active site of SrtC-2 as obtained from cluster analysis. The sorting signal is shown in licorice representation with its backbone additionally drawn as a yellow tube. Some important sortase residues that interact with the sorting signal are shown in ball and stick representation with associated one letter code and residue number.

In the SrtC-2 + YPRTG complex (Fig. 5), a hydrogen bond interaction occurs between Asp^203^ in the β6/β7 loop and the hydroxyl group of the sorting signal tyrosine (Table 1). In addition, Glu^200^ in the β6/β7 loop forms multiple hydrogen bonds with the sorting signal arginine, while Glu^130^, which is in a short α-helix between the β2 and β3 strands, interacts with both the arginine and threonine. Clustering analysis reveals a single major cluster containing 58% of the trajectory data. As observed previously for SrtC-1, several other clusters that are not far in RMSD distance were also obtained in addition to the major cluster. Structurally, the backbone conformation of the sorting signal in this dominant cluster has a RMSD of 2.4 Å from the representative of the main cluster of SrtC-1 + IPQTG (Table S1), indicating a pose that is similarly positioned to the one observed in SrtC-1. The sorting signal in the cluster representative of SrtC-2 + YPRTG complex adopts a more compact conformation, having an end to end distance of ~11 Å compared to ~14 Å for the SrtC-1 + IPQTG complex. This is likely due to the fact that the tyrosine sidechain of YPRTG requires more steric space than the isoleucine in IPQTG. In this conformation, the hydrophobic ring of the tyrosine tucks into a hydrophobic enclave, as was observed for the isoleucine residue of the SrtC-1 + IPQTG complex. However, the hydroxyl group points in the opposite direction and forms a hydrogen bond with the backbone of Asp^203^. The proline backbone interacts with the active site Arg^230^, while the arginine in the middle of the sorting signal is projected away from the active site. This gives it the conformational flexibility to form hydrogen bonds with Glu^200^ in the β6/β7 loop and Glu^130^ in the β2/β3 loop. Throughout the SrtC-2 + YPRTG simulation, the catalytic triad is well positioned for enzymatic activity, as the Cys^221^–Thr distance had an average value of 7.8 ± 3.4 Å, while the Arg^230^–Pro distance was 4.6 ± 2.4 Å.

**Figure 6:**
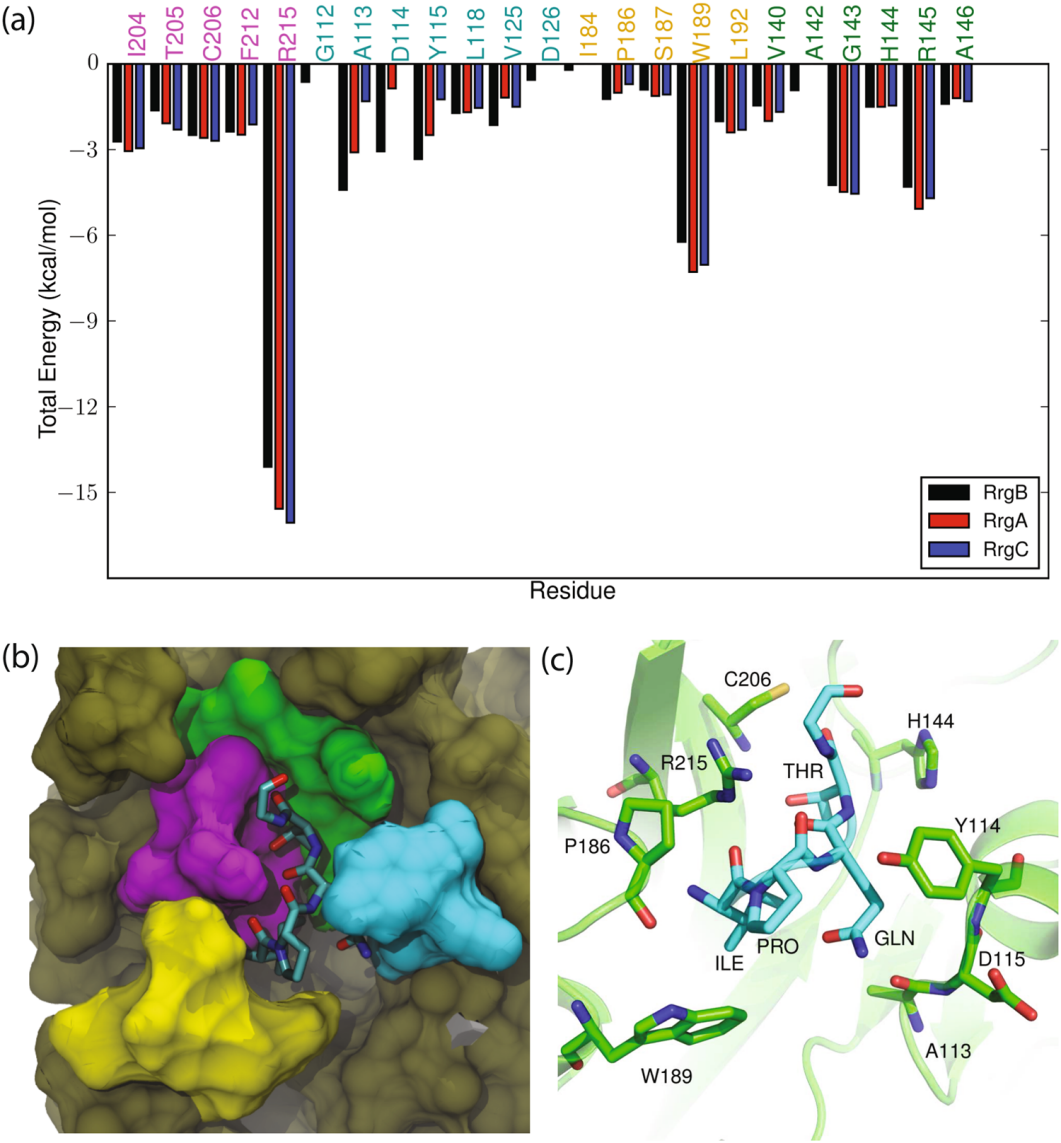
Interactions of Rrg sorting signals with SrtC-3: (**a**) Comparison of the total interaction energies between the RrgA (YPQTG), RrgB (IPQTG) and RrgC (VPDTG) sorting signals and the SrtC-3 protein. The four regions of SrtC-3 that interact with the sorting signal as described in the text are color coded with residue names in the graph, (**b**) Color coded surface representation of the aforementioned interaction regions and (**c**) Binding pose of the RrgB (IPQTG) sorting signal in the active site of SrtC-3 as obtained from cluster analysis. The sorting signal is shown in licorice representation with its backbone additionally drawn as a yellow tube. Some important sortase residues that interact with the sorting signal are shown in ball and stick representation with associated one letter code and residue number.

A smaller variety of hydrogen bonds were observed between the sorting signal and sortase in the SrtC-3 + IPQTG complex (Fig. 6). Of these SrtC-3 residues, hydrogen bonds between Arg^215^ and the sorting signal were the most prevalent. Interactions of Arg^215^ with the sorting signal isoleucine had the highest occupancy, although hydrogen bonds were also observed to the proline and glutamine. Further interactions were observed between Ala^113^, which is in the β2/β3 loop, with the glutamine. The hydrophobic sidechain of the isoleucine docked into a hydrophobic pocket, creating contacts similar to what was observed in the SrtC-1 + IPQTG complex. This, combined with tethering of the middle glutamine residue to Ala^113^ and the hydrogen bonds between the active site Arg^215^ and the proline and glutamine, securely fixes the sorting signal in the active site. Furthermore, the catalytic triad was well positioned throughout the simulation, with a Cys^206^–Thr distance of 4.1 ± 0.5 Å and a Arg^215^–Pro distance of 3.6 ± 0.9 Å. Cluster analysis indicated that the association of SrtC-3 with IPQTG is dominated by a single cluster which makes up nearly 100% of the analyzed 1.0 μs MD data. The conformation of the sorting signal in this complex is compact, similar to that of SrtC2 + YPRTG, which is reflected in the low RMSD difference of 2.1 Å between the dominant clusters reported in Table S1.

### Conformation of the Lid Region in the Presence of the Rrg Sorting Signal Peptides

**Figure 7:**
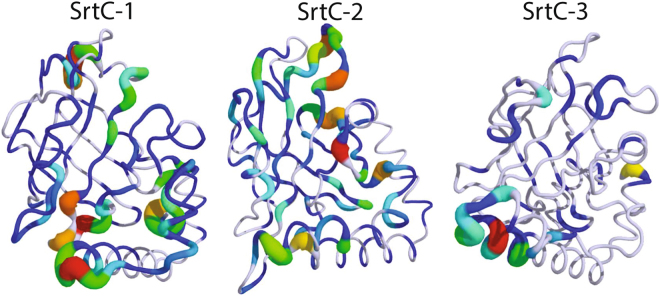
Models showing the Kullback-Liebler (KL) divergence between the apo and holo states of the sortase C enzymes. Thicker tubes indicate higher KL divergence and thus dynamical differences. White regions indicate statistically insignificant KL divergence while statistically significant divergence is graduated from blue to red.

**Figure 8:**
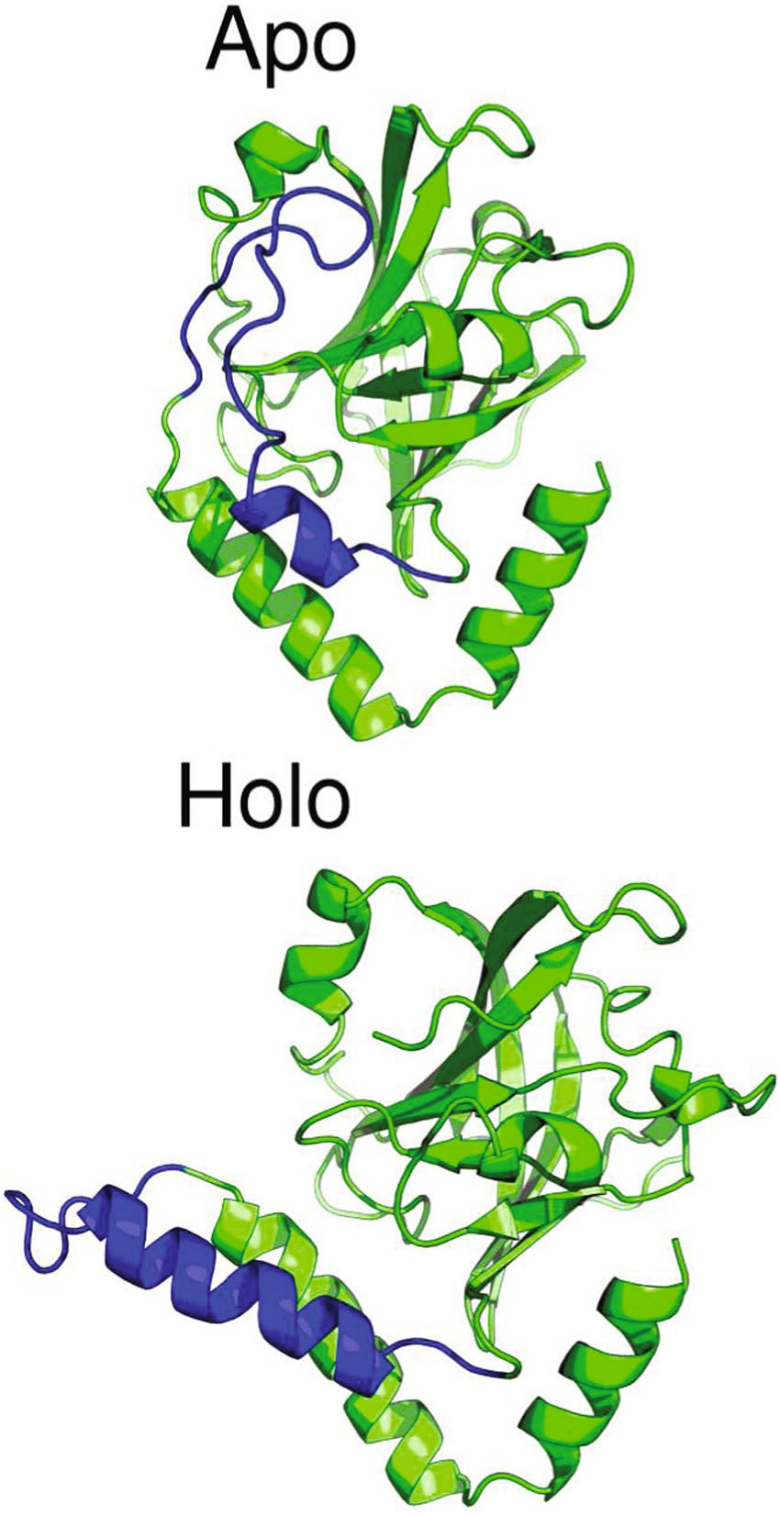
Comparison of the conformation of the lid region (blue color) of the SrtC-2 protein in the apo and holo states. While the region is a loop covering the active site in the apo state, in the presence of the sorting signal it is helical in structure, leaving the active site open for occupancy by the sorting signal.

**Figure 9:**
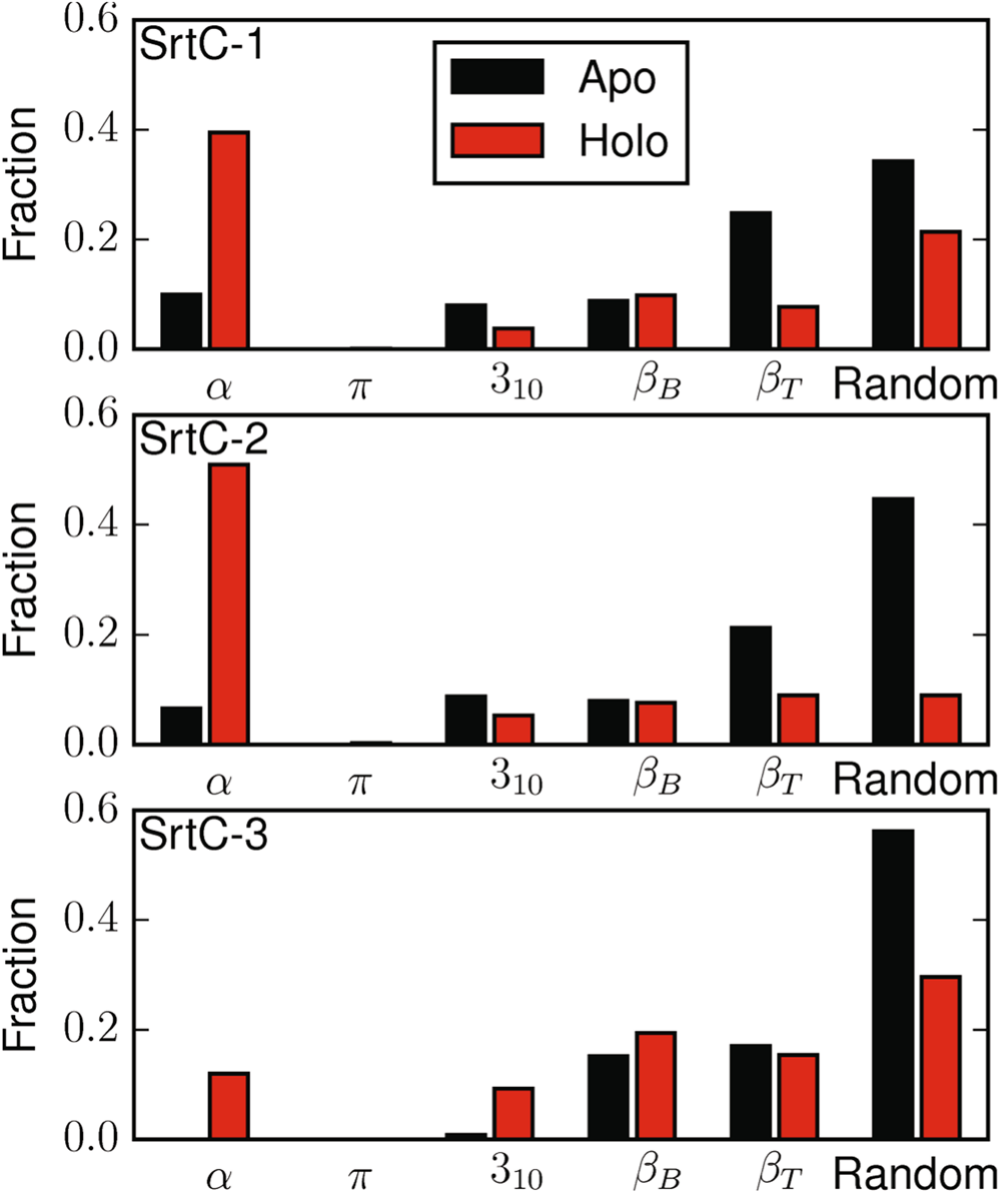
Secondary structure fractions of the lid region residues of SrtC-1, SrtC-2, and SrtC-3 in the apo and holo states. In each system substrate binding decreases the amount of random coil and increases the propensity for helix formation.

In each of the SrtC holo complexes, the sorting signal interacted with the conserved active site arginine and therefore disrupted the lid-anchoring hydrogen bonds between this residue and the lid. This, along with the insertion of the sorting signal, maintains the lid in an open state. The conformational dynamics of the lids in the bound state were compared to the apo state through a Kullback-Liebler (KL) divergence calculation between the backbone dihedral angles of each sortase in the presence and absence of a bound sorting signal (Fig. 7). Unsurprisingly, the analysis shows that the lid and the top portion of the β6/β7 loop are the regions that differ the most between the apo and holo states of the proteins. This reflects the observation that the lid remains open during substrate binding, in contrast with the apo state in which the lid region mostly adopts a loop structure that is tucked into the active site by salt-bridge and other interactions. As an example, this difference in lid conformation is further illustrated in Fig. 8 for SrtC-2 and Fig. 9 for all systems. In the case of SrtC-1 (Figure S2), two partial helices form whereas in SrtC-2 the lid region forms one long helix. In SrtC-3 (Figure S3), a similar loop to helix transition occurred but only resulted in the formation of a short helix. A longer helix did not form, even when the simulation was extended to over 4 μs. In all cases, the lid helici(es) are adjacent to the N-terminal α-helix as shown in Fig. 8 and contain the anchoring residues that are tucked in the active sites in the apo state. It should be noted that while the flanking regions (especially in the apo state) are in a random coil state as indicated by Fig. 9, the RMSF values are around 3 Å or less, therefore, the dynamics does not reflect large conformational changes.

These open conformations with the lid forming additional helices is in agreement with recently reported structures of *Streptococcus suis* (SsSrtC-1) and *Streptococcus agalactiae* (SagSrtC-1) which show open lid conformations where the lid transforms into a helix^50,51^.

### Energetics of Sortase C – Sorting Signal Binding

#### Decomposition of Force-field Interaction Energies

To explore the energetic basis for Sortase C pilin selectivity, we carried out an energy decomposition analysis on our multiple trajectory data (see Methods) for each of the nine potential Sortase C/sorting signal combinations. This analysis was used to determine the interactions that lead to sorting signal specificities by the three SrtC enzymes. Endpoint MM/GBSA analysis (Tables S2 to S4), which can provide a qualitative view of binding, were also carried out and are described in the SI. Results of MM/PBSA calculations follow the same trend in binding energy estimates (data not shown). While MM/GBSA did not produce results that ranked the SrtC/pilin binding selectivities in agreement with the more accurate alchemical calculations describe below, the analysis shows that the Rrg sorting signals experienced different molecular environments when bound to the various sortase C proteins.

The results of the decomposition analysis for the SrtC-1 complexes show that four main groups of residues contribute to binding (color coded in Fig. 4, and Table S5). First are the active site residues (Leu^191^, Cys^193^ and Arg^202^). Particularly, hydrogen bonds between the sidechain of Arg^202^ and the sorting signal backbone provides up to ~11 kcal/mol of stabilization for IPQTG binding. The next group of residues include Thr^129^, His^131^ and Thr^132^ which are located on the β4 strand and the loop following it. These contribute significantly to complex formation, but in most cases, have a higher magnitude for the RrgA and RrgB than the RrgC sorting signal. The likely reason for this is that the larger bulk of the isoleucine and tryptophan residues of IPQTG and YPRTG places the ends of the sorting signal in better contact with these residues. The next group includes residues Thr^174^, Asn^175^, and Leu^160^, situated in the β6/β7 loop, which have minimal interaction energies with the sorting signals. The final group consists of residues Thr^100^, Leu^105^, and Glu^172^, which are located on various loops surrounding the active site. Interactions with this last group of residues are mostly due to the middle residue of the sorting signal which points out of the active site into solvent.

This last set of interactions behave differently depending on the sorting signal. For the SrtC-1 + YPRTG (RrgA) complex, the positive arginine in the middle of the sorting signal interacts favorably with Glu^172^. However, doing this brings it into the proximity of the positively charged active site Arg^202^, and consequently unfavorable interactions. Specifically, the sorting signal middle arginine residue has electrostatic interaction energies of −12 and +22 kcal/mol with Glu^172^ and Arg^202^, respectively. When this arginine interacts with the negatively charged Glu^172^ that is located at the edge of the β6/β7 loop, it encounters unfavorable steric interactions with Arg^202^. However, these interactions are negated by the nonpolar solvation energies of +10 and −22 kcal/mol for interaction of this arginine with Glu^172^ and Arg^202^, respectively. These interactions are different for the negatively charged aspartic acid in the middle residue of the VPDTG (RrgC) sorting signal. Specifically, the electrostatic interaction energies are +11 and −26 kcal/mol with Glu^172^ and Arg^202^, respectively, while solvation energies are −11 and +26 kcal/mol. Interestingly, no interactions of such magnitude are observed for the SrtC-1 + IPQTG (RrgB) complex. Therefore, this analysis indicates that the charged RrgA and RrgC sorting signals experience electrostatic interactions (repulsion and attraction) that are not experienced by RrgB when bound to SrtC-1. Given that some of these interactions are screened by solvent, the implicit model used here may not accurately model these specific solvation effects, which may produce artifacts in the magnitudes of the binding free energies estimated here. To verify if this was the case, we analyzed the explicit solvent trajectories for the three complexes. This analysis shows that the arginine of YPRTG (RrgA) has on average 0.5 hydrogen bonds with water molecules. This means that it is likely that its unfavorable electrostatic interactions are not completely screened as predicted by implicit solvent analysis above. For example, the +22 kcal/mol steric interaction with Arg^202^ might not be completely counteracted by the nonpolar solvation energy. On the other hand, the negative aspartic acid in VPDTG (RrgC) is on average solvated by 1.8 hydrogen bonds. Thus, while it would have energetically benefited from direct sidechain interactions with Arg^202^, it is mostly screened from these favorable contacts. The OE1 group of the glutamine residue of IPQTG (RrgB) has on average 1.3 hydrogen bonds with solvent, however, its NE hydrogen groups are only partially solvated (0.3 hydrogen bonds), allowing it to hydrogen bond with ASN^199^ as previously described.

Like the SrtC-1 complexes, four regions contribute the most to the interactions between the sorting signals and SrtC-2 (Fig. 5 and Table S6). Active site residues (Cys^221^, Arg^230^), residues adjacent to the active site (His^159^, Thr^157^), the top section of the β6/β7 loop (Leu^140^, Asp^203^, Phe^204^), and residues in the β2/β3 short α-helix adjacent to the active site (Thr^128^, Ser^129^ and Glu^130^), interact the most with the sorting signals. Similar to SrtC-1, the charged middle residues of the YPRTG and VPDTG (RrgA and RrgC) sorting signals interact strongly with charged residues in the surrounding loops, however, in SrtC-2 these interactions are more favorable due to the positively charged arginine of YPRTG in this complex. This is largely due to the presence of several negatively charged sidechains including Glu^130^, Asp^131^, Glu^141^, Glu^200^, and Asp^203^. A specific salt bridge interaction between Glu^130^ and the YPRTG arginine provides approximately −44 kcal/mol of electrostatic stabilization to the complex, while the same residue disfavors complex formation with VPDTG by +24 kcal/mol. To avoid this unfavorable interaction, the middle aspartic acid residue in VPDTG points away into solvent and its backbone forms a hydrogen bond with Glu^130^. However, its short sidechain in still only ~4 Å away from Glu^130^ and thus experiences the previously mentioned repulsion. The net contribution of the Glu^130^/arginine interaction to the SrtC-2 + YPRTG complex is approximately −17 kcal/mol, the highest among all SrtC-2 complexes and is key to the selectivity of SrtC-2 to this sorting signal. Adding these interactions, as done in Tables S6, indicates that the YPRTG sorting signal is the preferred sorting signal for SrtC-2.

A similar grouping of residues as in the SrtC-1/2 complexes play key roles in the SrtC-3 complexes. For the SrtC-3 + YPRTG complex, the sidechain of the middle arginine points towards the residues in the short α-helix formed between β2 and β3. It interacts with Glu^114^ which shows a favorable electrostatic energy of -29 kcal/mol, although this is countered by a solvation energy of +27 kcal/mol. Such energy cancellation also occurs for its interaction with Asp^126^, with −18 versus +18 kcal/mol for electrostatic and polar solvation energies respectively. For the SrtC-3 + VPDTG complex, the negative sidechain of its middle residue interacts unfavorably with the negatively charged residues in the β2 and β3 helix as reflected in the electrostatics energies in Table S7. The glutamine in the IPQTG sorting signal points towards solvent but also interacts favorably with Tyr^115^ and the backbone of Ala^113^. Overall, the VPDTG sorting signal is least suited for this active site, which is reflected in the total energies of association.

#### Thermodynamic Integration

While MM/GBSA and energetic decompositions can provide information about the core interactions that govern the specificity and association in these complexes, approximations such as the lack of explicit solvent in the calculation limit its abilities to rigorously compute overall binding thermodynamics. To quantitatively characterize the energetics, we implemented thermodynamic integration (TI) calculations. We considered various TI data analysis methods using the Alchemical-analysis.py script implemented by Mobley and coworkers^47^. Table S8 shows an example output from the analysis of the TI data for the decharging step in the conversion of SrtC-1 + RrgB to SrtC-1 + RrgC. The trapezoid, BAR, and MBAR methods resulted in nearly identical results, and we therefore report the TI results below.

The average relative free energies for three sets of TI calculations between substrates and each SrtC isoform are given in Table 2. The results show that SrtC-1 preferentially binds to the IPQTG sorting signal of RrgB compared to RrgA, which is in agreement with experimental evidence that SrtC-1 and RrgB are the main sortase and pilin subunit. Similarly, TI agrees with experiments regarding the SrtC-1 preference of IPQTG (RrgB) over VPDTG (RrgC). TI analysis shows that SrtC-2 prefers the RrgA sorting signal (YPRTG) compared to that of RrgB, while both RrgB and RrgC are similarly preferred. This agrees with totals from decomposition analysis, as well as experimental results indicating that RrgA is the preferred first substrate for SrtC-2. Finally, SrtC-3 prefers RrgB to both RrgA and RrgC according to both TI and MM/GBSA analysis.

**Table 2:** Free energy (kcal/mol) of sorting signal transformations while in complex with the three SrtC enzymes calculated using thermodynamic integration.

Transformation	SrtC-1	SrtC-2	SrtC-3
RrgB to RrgA	1.4 ± 0.9	−2.9 ± 0.2	1.2 ± 0.5
RrgB to RrgC	2.5 ± 0.3	0.8 ± 0.4	2.7 ± 0.4

Standard errors of the mean obtained from three runs of each system are given in parenthesis.

## Discussion and Conclusion

In this work, we performed a series of MD simulations to characterize the molecular basis of the binding and selectivity of the three *S*. *pneumoniae* sortase C isoforms for Rrg sorting signals. First, our simulations reveal that the lid of all sortase proteins maintain an open conformation in the presence of the sorting signal peptides. The lid (or portions of it) relaxes into a helical structure that lies adjacent to the other N-terminal helices. This is in agreement with recently reported structures of *Streptococcus suis* (SsSrtC-1) and *Streptococcus agalactiae* (SagSrtC-1) which show open lid conformations where the lid transforms into a helix, as observed in our simulations^50,51^. Taken together, our results and these experiments suggests that the loop to helix transition of the lid upon substrate binding may be a general feature of SrtC enzymes.

Furthermore, our calculations show that the overall binding poses are similar for all SrtC and sorting signal combinations, although substrates bind to SrtC-2 and SrtC-3 in a slightly more compact conformation than SrtC-1. Additionally, the adopted structures are such that sorting signal/sortase contacts mirror many of the interactions that are present in the apo state between the lid and residues in and surrounding the active site. Specifically, the first two sorting signal residues are a proline and a hydrophobic residue which dock into the hydrophobic nook at the base of the β6/β7 loop. This is akin to the apo state where the residues in the lid cover the hydrophobic residues at the base of the β6/β7 loop. The backbone groups provide a replacement for the salt bridge interaction that existed between the lid and the conserved active site arginine. This, and other interactions between the backbone of the first sorting signal residue and residues adjacent to the active site, anchors the sorting signals into the active site and positions the threonine carbonyl for attack from the catalytic cysteine.

The middle residue in the Rrg sorting signals is pivotal to SrtC selectivity. In all cases studied here, this residue points away from the active site and creates contacts dictated by both its charge and length (reach) with surrounding loops that contain a number of charged residues. Specifically, RrgA contains a long and positively charged arginine, RrgB a neutral, polar, and slightly shorter glutamine, and RrgC a negatively charged and relatively small aspartic acid. For the SrtC-1 complexes, the RrgA sorting signal is situated such that its arginine can favorably interact with residues on the top of the β6/β7 loop, however this results in unfavorable steric interactions with the sidechain of the active site Arg^202^. The RrgB glutamine does not encounter any strong steric clashes and favorably interacts with Asn^199^. The sorting signal residues behaves similarly in SrtC-2 as in SrtC-1, however in this case the interactions are favorable for the RrgA sorting signal due to the formation of a salt-bridge to Glu^130^. This interaction helps shift the selectivity to RrgA in SrtC-2 over RrgB. Favorable interactions are also present for the RrgB sorting signal in complex with SrtC-3 compared to both RrgA and RrgC since the glutamine can favorably interact with the backbone of Ala^113^ while the RrgC glutamic acid can interact with the active site Arg^215^, resulting in Arg^215^ losing interactions with the sorting signal backbone.

Experiments point towards SrtC-1 as the primary sortase for pili formation in *S*. *pneumoniae* as it catalyzes the polymerization of the main RrgB pilin^18^. The alchemical free energy calculation results presented here support this conclusion, showing unfavorable free energy changes for transformations of the RrgB sorting signal into either of the two other pilin types. However, based on the magnitude of the free energy differences, the results indicate that SrtC-1 can potentially bind RrgA or RrgC sorting signals in the absence of RrgB. Indeed, according to work reported by Henriques-Normark and coworkers^16^, SrtC-1 (referred to as SrtB in their work) can catalyze the addition of RrgA and RrgC to pili. Additionally, Dessen and coworkers showed that SrtC-1 is at least capable of binding the RrgA sorting signal^27^. In their work, mutations to the two anchoring residues in the lid to glycines resulted in a drop of the melting temperature (T_m_) from 46 °C to 38 °C. However, addition of both RrgB and RrgA sorting signals to the mutated protein (SrtC-1-D58G/W60G) resulted in a rise in T_m_ from 38 °C to 43 °C, indicating that both peptides partially replace the role of the lid region and stabilize the sortase folded state.

Our alchemical calculations align with experimental results for the other SrtC isoforms. In particular, SrtC-2 shows a strong affinity for the YPRTG sorting signal of RrgA in comparison to the RrgB sorting signal according, which is in agreement with biochemical studies by Guilmi and coworkers^17^ which showed that SrtC-2 preferentially binds to RrgA and attaches it to the other pilins. Our calculations indicate that this preference is largely driven by the afore mentioned salt-bridge between the sorting signal arginine and the SrtC-2 Glu^130^ in the short helix between β2 and β3 strands. Calculations also show that SrtC-3 prefers the RrgB sorting signal compared to RrgA and RrgC. This is in accord with work by Neirs *et al*.^20^ that suggests that both SrtC-1 and SrtC-3 can bind RrgB, although SrtC-1 is the predominant RrgB sortase. Finally, our results indicate that none of the SrtC enzymes preferentially bind to the RrgC sorting signal, our results support the model be Shaik *et al*.^19^ which suggests that another sortase, SrtA, is primarily responsible for binding this motif and anchoring pili to the cell wall.

In conclusion, this work examined the interaction between the various Rrg sorting signals and SrtC proteins, and has revealed preferences that support and extended the emerging model of *S*. *pneumoniae* pili construction. Models of the sorting signal bound SrtC complexes show the sidechains of the first two residues complement hydrophobic residues around the active site, allowing the backbone of the sorting signal to interact with key residues in and around the active site, while projecting the middle sorting signal away from the catalytic triad. This middle residue interacts favorably (and unfavorably) with the residues in the surrounding loops and helix to increase or decrease binding affinities of the sorting signal to the SrtC. These results provide the first molecular level rationalization of the preferences of different sorting signals for SrtC and may be useful in designing compounds to limit the enzymatic activity of these important bacterial enzymes.

## Electronic supplementary material

Supplementary Information
