# Supplementary material for: Molecular Mechanisms of the Binding and Specificity of Streptococcus Pneumoniae Sortase C Enzymes for Pilin Subunits
Source: Sci Rep. 2017 Oct 13;7:13119. doi: 10.1038/s41598-017-13135-3 (PMC5640630; doi:10.1038/s41598-017-13135-3)
Supplement: Supplementary file 1 — Supplementary Information [file 41598_2017_13135_MOESM1_ESM.pdf]

Supporting Information

Molecular Mechanisms of the Binding and Specificity of *Streptococcus Pneumoniae* Sortase C Enzymes for Pilin Subunits

Emmanuel B. Naziga and Jeff Wereszczynski*

Department of Physics and Center for Molecular Study of Condensed Soft Matter, Illinois Institute of Technology, 3440 S Dearborn St, Chicago, IL 60616

* Email: jwereszc@iit.edu, Tel: +1 (312) 567 3322

MM/GBSA Analysis of Binding Energies

To explore the energetic basis for Sortase C pilin selectivity, two sets of free energy calculations were performed. To qualitatively asses the binding energies, and to decompose the thermodynamic basis for their differences, endpoint MM/GBSA analysis were used to estimate the free energy of association for each of the nine potential Sortase C/sorting signal combinations (Table S2). Overall free energy changes indicate that IPQTG (RrgB) forms the most stable complex with SrtC1, followed by YPRTG and then VPDTG (RrgA and RrgC). The energetic components are dominated by solvation (EGB+ESURF) and electrostatic (EEL+1-4 EEL) components. Solvation energies are more favorable for IPQTG and VPDTG in the complex compared to YPRTG, which is likely partly due to the hydrophobic nature of the isoleucine and valine sidechains. While the tyrosine of YPRTG is hydrophobic, its hydroxyl group is likely not well suited for the hydrophobic nook described in the previous section. In terms of the electrostatic contribution, VPDTG is the least favored in the SrtC1 complex, although all complexes have positive changes in electrostatic energies. The total Van der Waals (VDW) component (VDW + 1-4 VDW) of the energy is negative for all complexes and is roughly proportional to the size of the sidechain group of the first sorting signal residue. Specifically, the VDW energy is -39 kcal/mol, -44 kcal/mol and -34 kcal/mol, for the IPQTG, YPRTG and VPDTG complexes, respectively.

For SrtC-2, MM/GBSA analysis indicates that IPQTG (RrgB) is the most preferred substrate, followed by the YPRTG (RrgA) and then VPDTG (RrgC), which is the same ordering as for the SrtC-1 complexes (Table S3). However, the contributions from specific energy components differ from what was observed for SrtC-1. For the SrtC-2+IPQTG complex, electrostatics, VDW and solvation energies are almost of equal magnitude, with the first two being favorable while the latter is unfavorable. While the electrostatic component is large and favorable (-168 kcal/mol) for SrtC-2+YPRTG, the solvation energy is large and unfavorable (+170 kcal/mol) and the VDW energy is slightly favorable at -31 kcal/mol. The electrostatic energy for the VPDTG complex is the reverse of the case for YPRTG with large and positive electrostatic energy (+202 kcal/mol) and a large and negative solvation energy (-190 kcal/mol). This difference is largely attributed to the opposite charges of the arginine (positive) and aspartic acid (negative) sidechains for the YPRTG and VPDTG sorting signals.

For the SrtC-3 complexes, only IPQTG (RrgB) binding is energetically viable based on MM/GBSA analysis, having favorable electrostatic, solvation and VDW energies (Table S4). While the complex between SrtC-3 and YPRTG (RrgA) is favored by electrostatic interactions, its formation is negated by solvation energy, leading to an overall positive energy of association. The opposite is observed for the VPDTG (RrgC) sorting signal. Although the electrostatic component of the energy is unfavorable, the solvation is favorable, however, its magnitude is not sufficient to lead to complex formation. This correlates with the different charges of the sidechain of their middle residue. Interestingly, the VDW component is unfavorable for both YPRTG and VPDTG complexes.

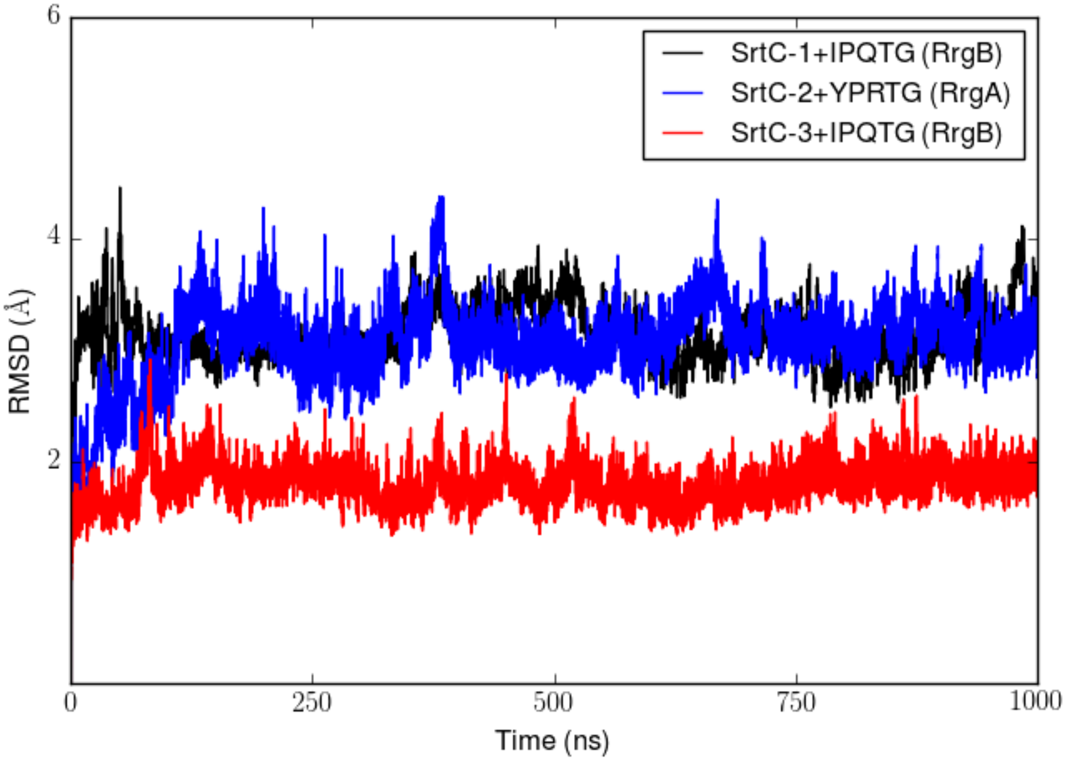

Figure S1: Root mean squared deviations of the SrtC-1+RrgB, SrtC-2+RrgA and SrtC-3+RrgB complexes.

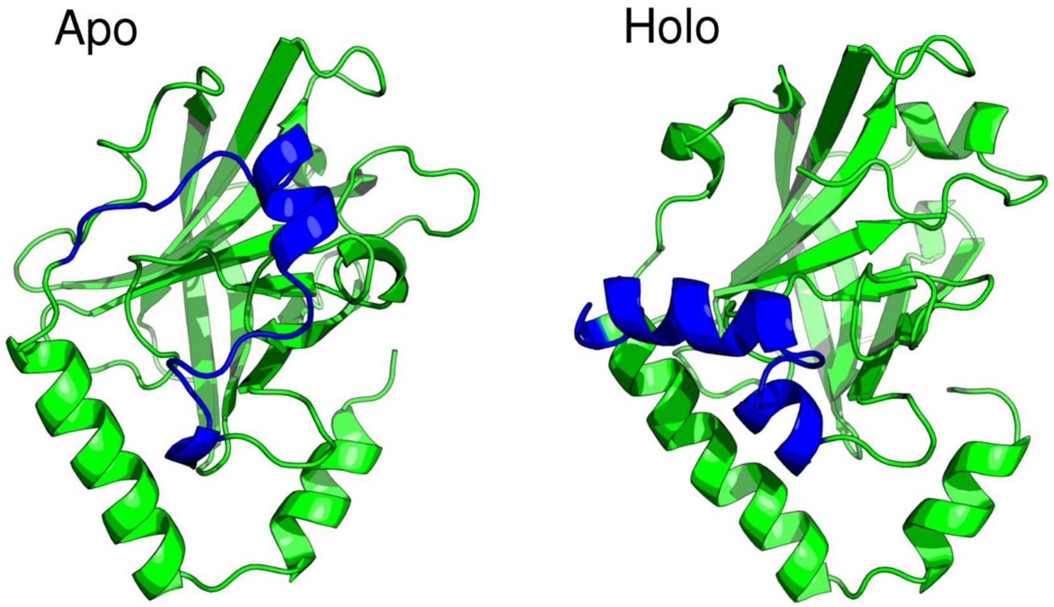

Figure S2: Comparison of the conformation of the lid region (blue color) of the SrtC-1 protein in the apo and holo states. While the region is a loop covering the active site in the apo state, in the presence of the sorting signal it is helical in structure, leaving the active site open for occupancy by the sorting signal.

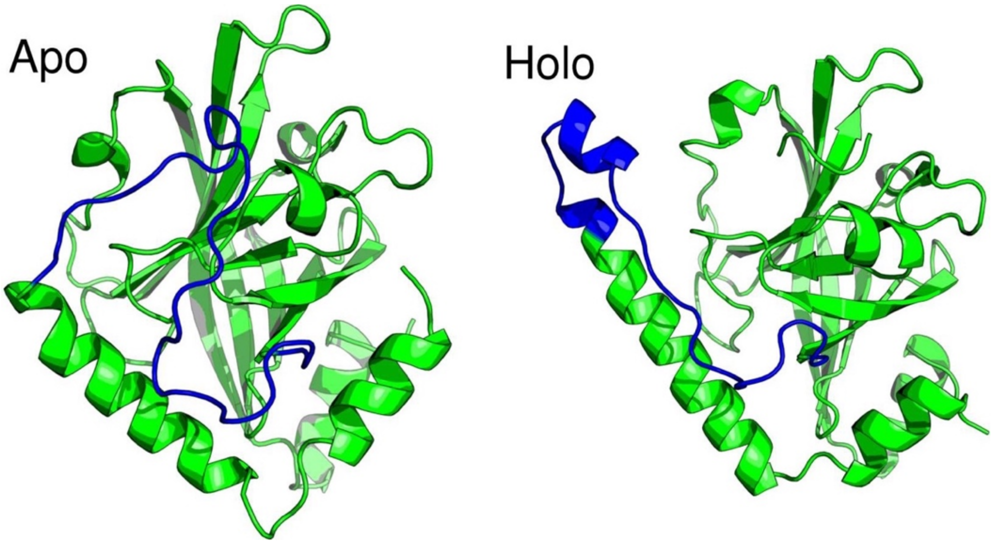

Figure S3: Comparison of the conformation of the lid region (blue color) of the SrtC-3 protein in the apo and holo states. While the region is a loop covering the active site in the apo state, in the presence of the sorting signal it is helical in structure, leaving the active site open for occupancy by the sorting signal.

Table S1: Backbone root mean squared displacement (RMSD) between the cluster representatives of the SrtC complexes. Two major clusters were obtained for the SrtC-1+IPQTG, while one cluster was obtained for SrtC-2+YPRTG and SrtC-3+IPQTG complexes.

	SrtC-1		SrtC-2	SrtC-3
Clusters	1	2	1	1
1	0	1.3	2.4	2.7
2	1.3	0	2.6	2.6
SrtC-2				
1	2.4	2.6	0	2.1

Table S2: MM/GBSA free energies (kcal/mol) of association between SrtC-1 and the RrgA (YPRTG), RrgB (IPQTG) and RrgC (VPDTG) sorting signals.

Energy Component	IPQTG		YPRTG		VPDTG	
	Mean	Std. Err.	Mean	Std. Err.	Mean	Std. Err.
Bond	-43.1	0.9	-41.4	1.0	-43.8	0.9
Angle	-5.3	1.4	-3.2	1.4	-4.5	1.4
Dihedral	-4.1	0.8	-4.5	0.9	1.9	0.8
VDW	-23.6	0.9	-29.4	0.9	-19.0	0.9
EEL	330.6	4.5	236.5	4.4	456.5	4.5
1-4 VDW	-15.2	0.5	-14.8	0.5	-13.9	0.5
1-4 EEL	-87.7	1.7	-86.0	1.7	-53.9	1.8
EGB	-225.3	3.7	-122.0	3.7	-385.7	3.7
ESURF	6.5	0.1	6.1	0.1	7.5	0.1
EGB+EEL	105.3	5.8	114.5	5.7	70.8	5.8
ESURF+VDW	-17.1	0.9	-23.3	0.9	-11.5	0.9
ΔG Gas	151.6	4.5	57.4	4.4	323.3	4.3
ΔG Solv.	-218.8	3.7	-116.3	3.7	-378.2	3.7
ΔG Total	-67.2	1.9	-58.9	1.9	-54.8	1.9

Table S3: MM/GBSA free energies (kcal/mol) of association between SrtC-2 and the RrgA (YPRTG), RrgB (IPQTG) and RrgC (VPDTG) sorting signals.

Energy Component	IPQTG		YPRTG		VPDTG	
	Mean	Std. Err.	Mean	Std. Err.	Mean	Std. Err.
Bond	-46.0	1.0	-45.7	1.0	-46.7	0.9
Angle	-10.2	1.5	-15.0	1.5	-16.5	1.4
Dihedral	4.5	0.9	9.8	0.9	8.8	0.8
VDW	-25.6	0.9	-18.0	0.9	-17.3	1.0
EEL	8.6	5.5	-136.2	5.6	248.6	5.8
1-4 VDW	-12.4	0.5	-10.7	0.5	-12.4	0.5
1-4 EEL	-50.9	1.8	-33.3	1.8	-46.6	1.8
EGB	41.2	4.7	164.8	4.9	-197.7	5.0
ESURF	5.5	0.1	6.6	0.1	7.4	0.1
EGB+EEL	49.8	7.2	28.6	7.4	50.9	7.7
ESURF+VDW	-20.1	0.9	-11.4	0.9	-9.9	1.0
ΔG Gas	-131.9	5.4	-249.2	5.6	118.0	5.7
ΔG Solv.	46.7	4.7	171.4	4.9	-190.4	5.0
ΔG Total	-85.2	2.1	-77.8	2.0	-72.4	2.0

Table S4: MM/GBSA free energies (kcal/mol) of association between SrtC-3 and the RrgA (YPRTG), RrgB (IPQTG) and RrgC (VPDTG) sorting signals.

Energy Component	IPQTG		YPRTG		VPDTG	
	Mean	Std. Err.	Mean	Std. Err.	Mean	Std. Err.
Bond	30.3	1.0	12.9	1.7	11.9	1.0
Angle	7.8	1.5	21.6	2.4	20.1	1.4
Dihedral	7.2	0.9	3.1	1.5	7.9	0.9
VDW	-47.1	0.9	10.4	1.5	5.7	0.9
EEL	24.2	5.1	-215.5	8.7	277.3	5.1
1-4 VDW	-17.6	0.5	50.6	0.9	50.3	0.5
1-4 EEL	-32.8	1.7	-41.7	3.0	-41.7	1.7
EGB	-15.5	4.2	229.2	7.2	-251.3	4.3
ESURF	-0.9	0.1	-1.3	0.1	-1.0	0.1
EGB+EEL	8.7	6.6	13.7	11.2	26.0	6.7
ESURF+VDW	48.0	0.9	9.1	1.5	4.7	0.9
ΔG Gas	-28.1	4.9	-158.5	8.3	331.7	5.0
ΔG Solv.	-16.4	4.2	227.9	7.2	-252.3	4.3
ΔG Total	-44.5	2.0	69.4	3.3	79.7	1.9

Table S5: Totals of decomposed interaction energies (kcal/mol) between SrtC-1 and the RrgA (YPRTG), RrgB (IPQTG) and RrgC (VPDTG) sorting signals. Calculated standard error of the mean is of the order of 0.1 kcal/mol for all energy components.

Energy Component	IPQTG	YPRTG	VPDTG
	Mean	Mean	Mean
VDW	-21.9	-23.4	-17.5
EEL	-30.9	-91.2	29.8
EGB	15.9	73.4	-41.4
ESURF	-15.9	-16.9	-12.9
EGB+EEL	-15.0	-17.8	-11.6
ESURF+VDW	-37.8	-40.3	-30.4
ΔG Total	-53.3	-58.1	-42.2

Table S6: Totals of decomposed interaction energies (kcal/mol) between SrtC-2 and the RrgA (YPRTG), RrgB (IPQTG) and RrgC (VPDTG) sorting signals. Calculated standard error of the mean is of the order of 0.1 kcal/mol for all energy components.

Energy Component	IPQTG	YPRTG	VPDTG
	Mean	Mean	Mean
VDW	-17.0	-20.5	-18.1
EEL	-21.6	-119.3	56.9
EGB	11.4	97.0	-16.6
ESURF	-12.9	-16.3	-13.2
EGB+EEL	-10.2	-22.3	40.3
ESURF+VDW	-29.9	-36.8	-31.3
ΔG Total	-40.2	-59.1	-41.1

Table S7: Totals of decomposed interaction energies (kcal/mol) between SrtC-3 and the RrgA (YPRTG), RrgB (IPQTG) and RrgC (VPDTG) sorting signals. Calculated standard error of the mean is of the order of 0.1 kcal/mol for all energy components.

Energy Component	IPQTG	YPRTG	VPDTG
	Mean	Mean	Mean
VDW	-22.2	-21.5	-20.1
EEL	-49.1	-173.8	72.3
EGB	30.3	152.3	-89.9
ESURF	-14.9	-15.7	-12.9
EGB+EEL	-18.8	-21.5	-17.6
ESURF+VDW	-37.1	-37.2	-30.0
ΔG Total	-61.1	-63.6	-56.5

Table S8: Thermodynamic integration decharging free energies (kcal/mol) for the various states during the conversion of RrgB (IPQTG) to RrgC (VPDTG) sorting signals when bound to SrtC-1 in water as determined by various analysis methods. The standard errors are given in parenthesis.

States	TI	TI-CUBIC	DEXP	IEXP	BAR	MBAR
0--1	16.5(0.03)	16.5(0.03)	16.8(0.04)	16.2(0.03)	16.5(0.03)	16.1(0.03)
1--2	15.7(0.02)	15.7(0.03)	16.1(0.03)	15.4(0.03)	15.7(0.02)	15.5(0.03)
2--3	14.9(0.02)	14.5(0.03)	15.2(0.03)	14.6(0.03)	14.9(0.02)	14.8(0.03)
3--4	14.1(0.02)	14.1(0.03)	14.4(0.03)	13.8(0.03)	14.1(0.02)	14.2(0.03)
4--5	13.5(0.02)	13.5(0.02)	13.6(0.03)	13.3(0.03)	13.5(0.02)	13.5(0.03)
5--6	13.0(0.02)	13.0(0.02)	13.2(0.03)	12.8(0.02)	13.0(0.02)	13.0(0.02)
6--7	12.5(0.02)	12.5(0.02)	12.7(0.03)	12.3(0.03)	12.5(0.02)	12.5(0.02)
7--8	12.0(0.02)	12.0(0.02)	12.2(0.03)	11.8(0.03)	12.0(0.02)	12.1(0.02)
8--9	11.5(0.02)	11.5(0.02	11.7(0.03)	11.2(0.03)	11.5(0.02)	11.7(0.02)
9--10	10.9(0.02)	10.9(0.02)	11.1(0.03)	10.7(0.02)	10.9(0.02)	11.3(0.02)
Total	134.6(0.09)	134.6(0.09)	137.0(0.10)	132.2(0.09)	134.6(0.07)	134.6(0.08)
